# Heteroresistance in *Mycobacterium tuberculosis*: from molecular mechanisms and advanced detection to clinical implications

**DOI:** 10.3389/fcimb.2026.1894646

**Published:** 2026-07-22

**Authors:** Shibo Liu, Dan Chen, Juanjue Zhang, Mingli Zhu

**Affiliations:** Open Laboratory, Hangzhou Xixi Hospital, Hangzhou Sixth People’s Hospital, Hangzhou, Zhejiang, China

**Keywords:** clinical implications, detection, heteroresistance, mechanisms, *Mycobacterium tuberculosis*

## Abstract

Drug resistance in *Mycobacterium* species, particularly *Mycobacterium tuberculosis*, remains a protracted and severe challenge to global public health. Recent studies have revealed that heteroresistance, serving as a critical precursor stage to full resistance, plays a pivotal role in treatment failure and disease relapse. Heteroresistance is characterized by the co-existence of susceptible and resistant subpopulations within a single bacterial population; however, due to the low proportion of the resistant subpopulation, it is highly susceptible to being missed in routine clinical diagnosis. This review summarizes the genetic and phenotypic mechanisms underlying the emergence of heteroresistance in *Mycobacterium tuberculosis*, evaluates the utility of various molecular detection technologies in identifying heteroresistance, and discusses its impact on clinical treatment regimen adjustments and the evolutionary trajectory of drug resistance. Ultimately, this review aims to provide a theoretical foundation for optimizing individualized, precision treatment for patients infected with *Mycobacterium tuberculosis*.

## 1 Introduction

*Mycobacterium tuberculosis* (Mtb) is a prominent member of the *Mycobacterium tuberculosis* complex (MTBC) and characteristically exhibits a slender, slightly curved rod-shaped morphology. Owing to its distinctive cell wall exceptionally rich in lipids and mycolic acids, this pathogen possesses the signature property of acid-fast staining, and is thus classically designated as an acid-fast bacillus, serving as the primary pathogen responsible for human tuberculosis (TB). Following the end of the COVID-19 pandemic, the incidence of TB has exhibited an upward trend. TB caused by Mtb has emerged as the leading infectious disease causing death, second only to COVID-19, particularly among immunocompromised populations (Mao et al., 2025). More importantly, according to the latest reports from the World Health Organization (WHO), the proportions of multidrug-resistant (MDR) and extensively drug-resistant (XDR) TB continue to rise, implying that the clinical management and treatment of tuberculosis will become increasingly challenging (Farhat et al., 2024).

Previously, it was widely accepted that drug resistance primarily originated from full resistance driven by fixed mutations. However, in recent years, a growing body of evidence indicates that heteroresistance (HR) is prevalent among clinical isolates and frequently represents an early or transitional stage in the evolution of drug resistance (Xu et al., 2025). HR is defined as the co-existence of drug-susceptible and drug-resistant subpopulations within a single monoclonal bacterial population (where the frequency of the resistant subpopulation is typically <50%, or even below 1%), resulting in an overall phenotype characterized as “subpopulation-mediated intermediate resistance.” This state represents a critical evolutionary phase, acting as a “gray zone” between full susceptibility and fixed, high-level resistance. This phenomenon was first described in *Haemophilus influenzae* and has since been identified across a wide range of bacterial species, most notably Mtb (Al-Shebiny et al., 2023; Liu et al., 2025; Moreira-Sousa et al., 2026).

To accurately elucidate the heteroresistance landscapes of Mtb within the host, a stringent conceptual differentiation among several core biological phenomena is imperative. Genetic heteroresistance refers to a dynamic phenomenon characterized by the intra-host coexistence of drug-susceptible wild-type and resistant subpopulations within a monoclonal bacterial population derived from a single common ancestor (Dewachter et al., 2019). Mixed infection specifically refers to an infection status wherein two or more genotypically distinct strains coexist simultaneously within a single host (Sobkowiak et al., 2018). Shifting to the non-heritable dimension, phenotypic tolerance represents a transient, population-wide capacity of bacteria to survive exposure to supra-therapeutic concentrations of bactericidal drugs due to environmentally induced metabolic deceleration (e.g., hypoxia or nutrient starvation); this survival capacity is fully reversible upon drug withdrawal and occurs in the absence of genomic mutations. Meanwhile, persistence is characterized by a microscopic, dormant, or metabolically stagnant subpopulation of “persister cells” (typically <1%) within a largely susceptible population, which arises from stochastic phenotypic heterogeneity and manages to endure lethal antibiotic onslaughts (Brauner et al., 2016). Ultimately, the progressive accumulation of the resistant fractions within these heteroresistant strains drives the evolutionary transition toward “full resistance” (Xu et al., 2025).

HR is commonly present in Mtb isolates worldwide, but its frequency varies among different anti tuberculosis drugs. Multiple studies have demonstrated that the incidence of HR against anti-tuberculosis drugs ranges from 4.3% to 57%, with the highest prevalence observed in fluoroquinolones (10%–38%), whereas the prevalence in rifampicin and isoniazid varies from 1% to 20% (Huo et al., 2020; Shivekar et al., 2020; Jouet et al., 2021; Ye et al., 2021). HR can arise from mixed infections or within-host clonal evolution. Its failure to be detected during clinical diagnosis and treatment can lead to treatment failure, relapse, and the selection of further drug resistance. This review provides a comprehensive overview of the dual genetic and phenotypic mechanisms driving HR in Mtb. It covers the evolution of diagnostic methods, from phenotypic susceptibility testing to highly sensitive molecular detection technologies, and analyzes the profound impact of HR on treatment outcomes. Furthermore, the review explores the persistent challenges and considerations for integrating HR detection into future clinical practice.

## 2 Mechanisms driving heteroresistance in Mtb

### 2.1 Molecular mechanisms

**Figure 1:**
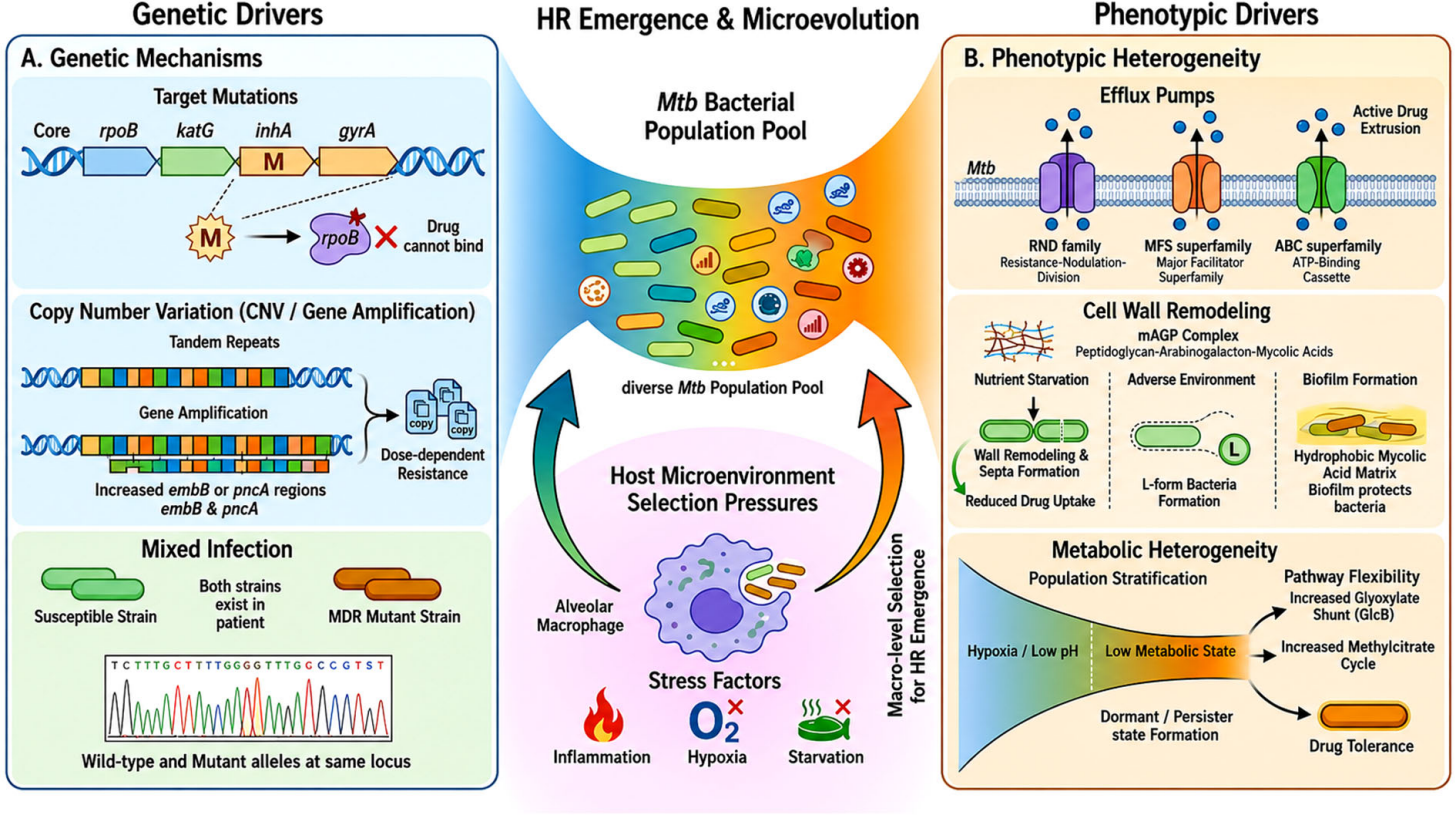
Multi-dimensional mechanisms underlying the development of heteroresistance in *Mycobacterium tuberculosis*. Heteroresistance (HR) is sculpted by a dynamic interplay between micro-level genetic variations and macro-level phenotypic adaptations. **(A)** Genetic mechanisms. HR can be driven by chromosomal alterations, including: (i) Target Mutations, where subclonal mutations in core genes (e.g., *rpoB*, *katG*) alter target proteins, preventing drug binding; (ii) Copy Number Variation (CNV/Gene Amplification), where amplification of regions like *embB* or *pncA* confers dose-dependent resistance; and (iii) Mixed Infection, where genetically distinct strains (e.g., susceptible and MDR mutant strains) co-exist within the same patient, presenting both wild-type and mutant alleles at the same locus during molecular diagnostics. **(B)** Phenotypic heterogeneity. HR is also mediated by non-heritable adaptations, such as: (i) Increased expression of *Efflux Pumps* (e.g., RND, MFS, ABC families), facilitating active extrusion of antimicrobial compounds; (ii) Cell Wall Remodeling, including septa formation under starvation, L-form bacteria formation in adverse environments, and biofilm cultivation in a mycolic acid matrix; and (iii) Metabolic Heterogeneity, where a subset of the population undergoes stratification to enter a hypometabolic dormant/persister state or exhibit pathway flexibility. The emergence and microevolution of these HR subpopulations are fundamentally driven by diverse Host Microenvironment Selection Pressures (e.g., inflammation, hypoxia, starvation), which screen for and promote resistant phenotypes from the diverse Mtb population pool.

The occurrence of HR in Mtb is essentially the result of the dynamic game between the genome homeostasis and environmental adaptability of the bacterial population in response to antibiotic selection pressure. At the genetic and molecular levels, HR is not driven by a single mechanism but rather exhibits multidimensional complexity. Specific gene mutations, copy number amplification of drug-resistant genes, and mixed infections with distinct genetic lineages can independently or synergistically shape the HR phenotype. These changes are subsequently transmitted across generations, thereby conferring a survival advantage under antibiotic pressure (**Figure 1**) (Jonsson, 2025).

#### 2.1.1 Target mutations

The essence of drug resistance in Mtb lies in the evolution of survival mechanisms to evade the bactericidal effects of drugs, with genomic mutations serving as the primary driver. Mutations in specific genes alter drug targets or compromise drug efficacy, enabling the bacteria to develop resistance and survive even during chemotherapy (Amukti et al., 2024). Studies have demonstrated that mutations in genes such as *rpoB*, *katG*, *inhA*, and *gyrA* are the most frequently observed in Mtb; however, these mutations are often confined to a minority of subclones, which constitutes a common etiology of HR (de Assis Figueredo et al., 2020; Dixit et al., 2023). Beyond these, mutations in genes including *fabG1*, *embB*, *pncA*, *gyrB*, *rrs*, *eis*, *rpsL*, *atpE*, *Rv0678*, *pepQ*, *Rv1979c*, *rrl*, *rplC*, *ddn*, *fgd1*, *fbiA*, *fbiB*, and *fbiC* can also induce HR to various anti-tuberculosis drugs. Consequently, alterations across these 22 gene targets enable the prediction of Mtb resistance to 14 distinct drugs, namely rifampicin, isoniazid, ethambutol, pyrazinamide, moxifloxacin, levofloxacin, amikacin, capreomycin, kanamycin, streptomycin, bedaquiline, clofazimine, linezolid, and delamanid (Wu et al., 2022). This predictive capacity forms the cornerstone of molecular-based drug resistance testing for Mtb. Nevertheless, with the widespread adoption of molecular diagnostics, novel resistance-associated genes are continuously being identified. To standardize interpretation, the WHO published a comprehensive catalogue of Mtb mutations associated with drug resistance in 2021, incorporating 49 candidate resistance genes as a global standard, which was subsequently updated and expanded to 68 genes in 2023 (World Health Organization, 2023). Despite the extensive array of resistance loci included in the WHO catalogue, commercial molecular drug susceptibility testing (DST) assays cover only a limited number of diagnostic targets. As a result, a substantial risk of missed detection persists for HR driven by rare genetic mutations.

#### 2.1.2 Gene copy number variation

Early comparative genomic studies of Mtb revealed that tandem repeat sequences at dozens of loci within its genome frequently differed between laboratory strains and clinical isolates, suggesting that the Mtb genome is not static but rather undergoes phase variation (Supply et al., 2000). Subsequent research demonstrated that copy number variation of tandem repeats is a common modality of phase variation in Mtb, capable of generating subpopulations with distinct clinical phenotypes. The amplification of tandem repeats in certain drug-resistance-associated genes (such as regions related to *embB* and *pncA*) can lead to dose-dependent resistance, thereby driving the emergence of the HR phenotype (Silva and Khare, 2024). Concurrently, homologous recombination can occur between interspersed repeat sequences, generating “novel” genetic or regulatory structures within clonal organisms and dictating the evolutionary trajectory of Mtb (Stritt et al., 2025). This heterogeneous clonal population generated by gene copy number variations not only confers drug resistance—thereby enhancing survival probability within fluctuating drug environments—but also facilitates evasion of host immunity.

#### 2.1.3 Mixed infection

Mixed infection is another prevalent mechanism underlying the development of HR in Mtb, defined as the co-existence of strains exhibiting distinct patterns across two or more genetic loci (Gamberale et al., 2023). Mixed infections can simulate or promote the emergence of heteroresistant phenotypes. Based on the genetic relationships between the strains, these scenarios can be categorized into polyclonal mixed infections and monoclonal heteroresistance, which exhibit overlapping features yet possess fundamental distinctions. Polyclonal mixed infection refers to the simultaneous infection by two or more strains with entirely distinct genetic backgrounds. This may occur through reinfection, exposure to multiple circulating strains, or concurrent infection with susceptible and resistant strains within high-burden transmission environments, thereby reflecting strain diversity and dynamic transmission dynamics. Conversely, clonal heteroresistance arises when a single strain differentiates into resistant sub-populations within the host via *de novo* mutations, reflecting the microevolution of the bacteria themselves within the host macro-environment (Zheng et al., 2015; Kargarpour Kamakoli et al., 2017; Komakech et al., 2024). Although their underlying mechanisms differ, both scenarios frequently yield identical profiles in molecular assays (such as next-generation sequencing), where wild-type and mutant alleles coexist at the same genomic locus. Definitively differentiating this state of wild-type and non-wild-type coexistence necessitates additional genomic data, including lineage-specific markers, single-nucleotide polymorphism (SNP) distances, and strain-specific variation patterns (Keo, 2016; Rigouts et al., 2019). Similarly, this mixed infection status favors the long-term survival and transmission of drug-resistant strains, which are more prone to becoming the dominant population post-treatment, thereby completing the transition from heteroresistance to full resistance under prolonged antibiotic selective pressure (Ng et al., 2019). Nevertheless, from an evolutionary or genetic perspective, this macro-level population heterogeneity driven by mixed infections ultimately remains fundamentally attributable to alterations at the genetic level (Castro et al., 2021).

### 2.2 Phenotypic heterogeneity

Phenotypic Heterogeneity Distinct from HR driven by genetic mutation mechanisms, phenotypic heterogeneity refers to the phenomenon where genetically identical Mtb subpopulations exhibit divergent drug susceptibility as a mode of adaptation to dynamic environments, they serve as critical factors facilitating this phenotypic heterogeneity and potentially fostering environments conducive to the emergence, survival, or selection of heteroresistant sub-populations. These non-genetic factors encompass differential efflux pump expression, cell wall alterations, metabolic shifting, and host microenvironmental influences (**Figure 1**).

#### 2.2.1 Efflux pumps

Efflux pumps are intracellular structures, predominantly transmembrane proteins, that traverse the cell envelope to form channels capable of transporting a wide array of substrates—including antibiotics—into and out of Mtb cells. These systems play a critical role in the emergence of MDR and XDR strains (Abavisani et al., 2022). Unlike genetic mutation-induced alterations in bacterial drug susceptibility, efflux pump-mediated resistance is primarily triggered by the dysregulation of gene expression or altered protein functionality following antibiotic exposure. This leads to the active extrusion of various essential molecules, including antimicrobial compounds, out of the cell, resulting in decreased intracellular drug concentrations (Chimal-Muñoz et al., 2025; Sharma et al., 2025). This tolerance mechanism is classified as induced tolerance, meaning that efflux pumps are activated via gene expression regulation under drug pressure rather than stable drug resistance driven by genetic mutations (Goossens et al., 2020). Concurrently, the upregulation of efflux pumps is intimately linked to the formation of persistent cell sub-populations, exhibiting characteristics of heterotolerance (Parbhoo et al., 2022). Studies indicate that efflux pumps in Mtb comprise the Major Facilitator Superfamily (MFS), the Proteobacterial Antimicrobial Compound Efflux (PACE) family, the Multidrug and Toxic Compound Extrusion (MATE) family, the Small Multidrug Resistance (SMR) family, the ATP-Binding Cassette (ABC) superfamily, and the Resistance-Nodulation-Division (RND) family (Laws et al., 2022). Although the core scaffold architecture across these transporter families is evolutionary conserved, individual members within each family vary in their amino acid sequences. Consequently, the drug resistance profiles conferred by different transporter families vary accordingly. A comprehensive review revealed that the RND and SMR families can mediate resistance to isoniazid, rifampicin, ethambutol, streptomycin, and bedaquiline. Beyond these agents, the MFS family can additionally confer resistance to kanamycin and fluoroquinolones, while the ABC superfamily mediates resistance to rifampicin, isoniazid, kanamycin, and fluoroquinolones; conversely, the substrate profile of the MATE family is restricted to isoniazid and rifampicin resistance (Sharma et al., 2025).

#### 2.2.2 Cell wall

The cell wall of Mtb is an intricate network predominantly composed of peptidoglycan, arabinogalactan, and mycolic acids, collectively designated as the mAGP complex. The complex permeability barrier it creates precludes the effective penetration of multiple drugs, and this low permeability underpins the intrinsic resistance of Mtb to various antibiotics (Batt et al., 2020; Schami et al., 2023). Crucially, the Mtb cell wall is not a static structure but is modulated by the growth environment (Koh et al., 2022). Under nutrient starvation, the cell wall undergoes remodeling to form septa, a process accompanied by a significant reduction in bacterial drug uptake (Wu et al., 2016). Under more adverse environmental conditions, Mtb can even shed its cell wall to transition into L-form bacteria, which serves as a vital mechanism for its transient survival under antibiotic pressure (Chen et al., 2020; Lei et al., 2025). Furthermore, Mtb undergoes asymmetric cell division, wherein newly synthesized cell wall material is deposited asymmetrically at the bacterial poles. This spatial discrepancy in cell wall elongation leads to variations in growth rates, size, and macromolecular distribution between daughter cells, this spatial asymmetry in cell wall division creates favorable conditions for the emergence of heteroresistant sub-populations (Dulberger et al., 2020). Additionally, alterations in the relative proportions of different components within the cell wall matrix can modulate Mtb susceptibility to specific compounds; for instance, a reduction in cell wall arabinogalactan enhances the anti-tuberculosis activity of bedaquiline, a phenomenon primarily driven by increased drug uptake resulting from enhanced permeability following inner cell wall alterations (Li et al., 2022). Corroborating evidence indicates that drug-resistant Mtb (DR-Mtb) strains exhibit distinct differences in cell wall thickness, surface morphology, and internal composition compared to their drug-susceptible counterparts (Mehaffy et al., 2018). Moreover, Mtb can cultivate highly hydrophobic extracellular matrices composed of free mycolic acids to form biofilms *in vitro*, thereby resisting high doses of anti-tuberculosis drugs and facilitating the emergence of phenotypic HR (Allué-Guardia et al., 2021).

#### 2.2.3 Metabolic heterogeneity

Metabolic heterogeneity serves as a pivotal mediator of phenotypic HR in Mtb and constitutes a critical mechanism driving the development of phenotypic resistance under host-induced pressure. When bacterial populations are exposed to environmental stressors such as hypoxia, oxidative stress, osmotic fluctuation, nutrient deprivation, and low pH, certain subpopulations actively modulate their metabolic states to enter a hypometabolic “dormant” or “persister” state. This process encompasses a multitude of metabolic pathways, including those governing the utilization of glucose, glycerol, oleic acid, cholesterol, and various amino acids. This metabolic “stratification” within the bacterial population engenders disparate drug tolerance at the subpopulation level, only actively proliferating sub-populations are susceptible to bactericidal drugs, whereas metabolically inactive semi-dormant and completely dormant sub-populations exhibit drug tolerance (Costa Gouveia, 2017; Samuels et al., 2022). Untargeted metabolomic analyses have unveiled marked discrepancies between the intracellular and extracellular (culture supernatant) metabolomic profiles of clinical drug-susceptible strains and those of multidrug-resistant strains. These differentially expressed metabolites are predominantly enriched in nicotinic acid/nicotinamide metabolism, purine/pyrimidine metabolism, and arginine metabolism pathways—which are intrinsically linked to Mtb growth, physiological functionality, and host defense mechanisms, respectively—suggesting that acquired drug resistance can profoundly impact bacterial physiology via metabolic rewiring (Bobba and Khader, 2023; Wang et al., 2023).

Furthermore, the degree of Mtb reliance on specific metabolic circuits modulates its drug resistance. Utilizing transposon sequencing (Tn-seq), Carey et al. discovered that clinical isolates belonging to Lineage 2 exhibit significantly lower dependency on the glyoxylate shunt (specifically its key enzyme, malate synthase/GlcB) compared to the traditional laboratory reference strain H37Rv. This metabolic “flexibility” enables these clinical strains to survive despite the disruption of this pathway, thereby evading drug regimes targeting this specific axis (Carey et al., 2018). Additionally, the accumulation of intermediates from alternative metabolic processes, such as the methylcitrate cycle, can also augment resistance; the underlying mechanism involves the perturbation of Mtb respiratory activity and membrane bioenergetics, culminating in growth arrest and subsequent drug tolerance (Quinonez et al., 2022). Concurrently, specific sub-populations can manifest heightened tolerance to antibiotics through alterations in the tricarboxylic acid (TCA) cycle and its downstream electron transport chain activity, which induce oxidative stress within Mtb and this scenario potentially involves a dual mechanism of induced tolerance (driven by drug-induced metabolic remodeling) and heterotolerance (characterized by intra-population variations in metabolic states (Samuels et al., 2022; Lee et al., 2025). Moreover, upgrading the catalytic turnover activity of trehalose in Mtb can generate pre-resistance strains, which serve as a critical prerequisite facilitating the emergence of HR (Lee et al., 2025). In short, this heterogeneous distribution driven by metabolic flexibility allows hypometabolic subpopulations to survive lethal concentrations of antibiotics, posing a substantial hidden threat that leads to clinical treatment failure and subsequent secondary genetic resistance.

#### 2.2.4 Host microenvironment

The initial cascade of Mtb infection begins when Mtb bacilli contained within aerosols are inhaled into the respiratory tract, reach the lungs, and are subsequently engulfed by tissue-resident alveolar macrophages, crossing the epithelium to enter the interstitial tissue. A fraction of the viable bacilli is then translocated to the thoracic lymph nodes, where they proliferate before entering the bloodstream via the thoracic duct, leading to systemic dissemination and subsequent colonization at diverse anatomical sites (Yang et al., 2023b). Owing to its robust survival apparatus, Mtb can endure within a multitude of distinct microenvironments inside the host. Crucially, the marked heterogeneity of the host organs and microenvironments further drives the diversification of Mtb, thereby amplifying the probability of observing HR within a single tuberculosis patient. This intra-host Mtb diversity can theoretically stem from mixed infections involving multiple strains with distinct genetic backgrounds, within-host microevolution of a single infecting strain, or a combination of both (Ford et al., 2012). In extreme scenarios, studies profiling advanced tuberculosis patients have documented up to 50 consensus-level single nucleotide polymorphism (SNP) differences across samples harvested from multiple anatomical sites (Lieberman et al., 2016).

On one hand, Mtb has co-evolved with humans, a protracted evolutionary relationship that has prompted Mtb to fine-tune its genetic and epigenetic landscapes to adapt to diverse intra-host microenvironments (Modlin et al., 2026). On the other hand, the dynamic microenvironments forged during host-pathogen interactions subject distinct Mtb subpopulations to divergent intracellular conditions, thereby facilitating phenotypic diversification and heterogeneity (Subhash et al., 2025). In conclusion, microenvironment-associated stressors—including inflammation, hypoxia, and starvation—are intimately correlated with elevated bacterial mutation rates and the emergence of drug-resistant alleles within the genome, playing an indispensable role in the manifestation of intra-host Mtb heteroresistance phenotypes (Allué-Guardia et al., 2021).

## 3 Detection technologies for heteroresistance

### 3.1 Phenotypic drug susceptibility

Testing For decades, phenotypic drug susceptibility testing (pDST) has been acknowledged as the gold standard for diagnosing drug susceptibility. By monitoring the survival rate and metabolic inhibition of Mtb in environments containing anti-tuberculosis agents, this assay directly evaluates the replicative capacity of bacteria under antibiotic exposure, independent of the underlying genetic mechanisms (Kim, 2025). Evidence suggests that pDST can detect HR at a frequency as low as 1%, a threshold significantly superior to the average limit of detection (LOD) offered by whole-genome sequencing (WGS) and Xpert assays (Ismail et al., 2025). However, a paramount drawback of culture-based methods is their stringent biosafety requirements and protracted turnaround time. This is especially true for Mtb, where generating definitive results often necessitates several weeks or even months, inherently conflicting with the clinical imperative for rapid diagnosis (Zhu et al., 2021).

Furthermore, the critical concentrations of drugs designated in pDST profoundly influence the diagnostic sensitivity for Mtb heteroresistance. For instance, while the WHO recommends a critical concentration of 1.0 μg/mL for rifampicin in the Mycobacteria Growth Indicator Tube (MGIT) medium, studies have demonstrated that utilizing a lower threshold of 0.5 μg/mL facilitates the detection of low-level Mtb resistance that may carry clinical relevance. A comparable phenomenon has also been documented for fluoroquinolones. Collectively, these findings underscore that optimizing the critical concentrations utilized in pDST could substantially enhance the detection rate of HR in Mtb isolates (Rupasinghe et al., 2024; Thomas et al., 2024).

### 3.2 Molecular detection technologies

Although culture-based methods are traditionally deemed the gold standard for drug susceptibility testing, molecular assays offer rapid profiling of Mtb mutations associated with anti-tuberculosis drug resistance, drastically curtailing the protracted turnaround times inherent to pDST (Domínguez et al., 2016). Nonetheless, distinct molecular platforms exhibit divergent advantages and constraints regarding their capacity to detect Mtb heteroresistance.

#### 3.2.1 Xpert assays

As early as 2010, the WHO endorsed the utilization of the Xpert MTB/RIF assay for the rapid point-of-care diagnosis of tuberculosis and rifampicin resistance (World Health Organization, 2013). Currently, the Xpert portfolio dedicated to TB diagnostics comprises three assays: Xpert MTB/RIF, Xpert MTB/RIF Ultra, and Xpert MTB/XDR, which are extensively deployed across more than 120 countries (Huang et al., 2023). Prior research indicates that the original Xpert MTB/RIF assay can accurately identify rifampicin resistance only when the DR-Mtb subpopulation constitutes at least 40% of the total bacterial load within a specimen (Zetola et al., 2014). Conversely, the Xpert MTB/RIF Ultra assay substantially lowers this limit of detection (LOD). Initial validation studies conducted by the manufacturer established LOD values for specific mutations, such as 20% to 40% for L430P and H445N, and 5% to 10% for S450L; this heightened sensitivity is primarily attributable to the melting temperature (Tm) analysis-based readout deployed in MTB/RIF Ultra to differentiate between wild-type and mutant strains (Chakravorty et al., 2017). Correspondingly, alternative studies have demonstrated that for rifampicin HR, the LOD of GeneXpert MTB/RIF stands at 60%, whereas GeneXpert MTB/RIF Ultra achieves an LOD of 10%, a performance comparable to that of WGS (Danchuk et al., 2024). Nevertheless, conflicting reports caution that while certain probe patterns yielded by the Ultra assay are considered indicative of HR, the sensitivity and specificity of these patterns for diagnosing HR remain suboptimal, occasionally predisposing the assay to false-positive rifampicin resistance results (Ghebrekristos et al., 2025). Compared to Ultra, the Xpert MTB/XDR assay expands the diagnostic repertoire to include additional anti-tuberculosis agents, notably second-line injectable drugs. Evidence shows that Xpert MTB/XDR can detect isoniazid HR at a frequency of 10%, ethionamide, amikacin, and kanamycin HR at 50%, and capreomycin HR at 60%; regrettably, this platform has not yet achieved widespread accessibility in the majority of nations (Georghiou et al., 2021).

#### 3.2.2 Line probe assays

Line Probe Assays (LPAs) represent another suite of rapid resistance-diagnosing modalities recommended by the WHO. Early implementation of LPAs highlighted their intrinsic capability to detect HR, characterized by the simultaneous visualization of both wild-type and mutant bands on the strip (Daniel Mekonnen et al., 2015). Although the diagnostic target menu of LPAs has expanded from rifampicin and isoniazid to encompass multiple anti-tuberculosis drugs—including certain second-line agents—their LOD for HR has consistently hovered around 5% (Werngren et al., 2021; Ranjan et al., 2023; Thosani et al., 2023). Several investigators argue that the direct application of LPAs to clinical specimens could amplify the detection rate of HR, particularly among newly diagnosed patients with MDR-TB/rifampicin-resistant TB or isoniazid-resistant TB, thereby facilitating the formulation of individualized therapeutic regimens and preventing further transmission (Madhuri et al., 2015; Getahun et al., 2022). However, a large-scale, multicenter clinical evaluation revealed that while LPAs reliably detect drug resistance with low error rates, their sensitivity is highly contingent upon specific mutation types compared to targeted next-generation sequencing (tNGS). Consequently, LPAs may systematically overlook and fail to capture less common resistance mutations associated with rifampicin and isoniazid resistance (Carpi et al., 2025). Additionally, LPAs face certain limitations in detecting pyrazinamide HR, primarily driven by probe ambiguities (Driesen et al., 2018). Therefore, the direct clinical application of LPAs for capturing HR in patient specimens warrants more rigorous, extensive evaluation.

#### 3.2.3 Droplet digital PCR

Droplet Digital Polymerase Chain Reaction (ddPCR) capitalizes on microfluidic technology to partition a standard PCR mixture into a water-in-oil emulsion comprising thousands of nanoliter droplets. This partitioning enables thousands of independent, discrete PCR amplifications to transpire within a single reaction tube, thereby facilitating the absolute quantification of both wild-type and mutant DNA molecules in a specimen. Consequently, ddPCR offers unparalleled sensitivity and accuracy in measuring the proportions of drug-resistant subpopulations (Bessa-Gonçalves et al., 2024). A study investigating the application of ddPCR for identifying and quantifying HR within a TB cohort characterized by mixed infections demonstrated its capacity to detect mutant alleles at a frequency as low as 0.1% (Zheng et al., 2022). Its superior advantage—possessing a limit of detection well below the conventional 1% clinical threshold—has been validated across resistance testing for multiple anti-tuberculosis agents, including isoniazid and fluoroquinolones (Rigouts et al., 2019; Zhang et al., 2023). Clinical investigations evaluating sputum samples from newly diagnosed TB patients have shown that ddPCR successfully discriminates and quantifies both mutant and wild-type strains in clinical populations. This capacity allows for the timely interception of HR and MDR-TB, highlighting its potential as an efficacious tool for the early detection and management of drug-resistant tuberculosis (Aung et al., 2023).

#### 3.2.4 WGS

WGS enables the concurrent profiling of multi-locus variations alongside their respective variant allele frequencies (VAF). A VAF below 20% to 50% typically denotes that the drug resistance resides within a heterogeneous subpopulation (Shi et al., 2018). For standard-coverage WGS (50–100×), the theoretical detection limit for HR is approximately 5%–10%; however, most bioinformatic pipelines routinely implement a conservative 10% calling threshold to minimize false-positive calls. In contrast, deep sequencing (>1000×) can resolve variants present at frequencies of 0.1%–1%, with the calling threshold generally established at 1%–3% based on background error rates (Sandmann et al., 2020). Notably, recent studies have reported that integrating specialized variant-calling toolkits—which model run-specific error rates and position-specific sequencing biases—can push the detection floor below 0.05%, although its performance in specific clinical applications warrants further evaluation (Goossens et al., 2022).

Furthermore, implementing distinct error-correction modalities during the sequencing workflow can substantially augment the sensitivity of HR detection. Reports indicate that error-correction algorithms leveraging single-molecule overlapping reads significantly mitigate sequencing artifacts, enabling the robust detection of DR-Mtb subpopulations comprising as low as 0.1% of the total bacterial community (Wang et al., 2019). Additionally, a novel “sequencing-by-binding” chemistry has demonstrated the capacity to sequence well-characterized Mtb resistance mutations within the *katG* and *gyrA* genes with a sensitivity reaching 0.01%, entirely bypassing the need for additional computational error-correction steps (Allender et al., 2024). Collectively, these landmarks underscore the profound potential of WGS in identifying and quantifying low- to ultra-low-frequency HR subpopulations; nevertheless, the prohibitive costs of WGS, coupled with its intensive and complex bioinformatic interpretation, continue to constrain its widespread deployment in routine clinical settings.

#### 3.2.5 tNGS

tNGS has garnered substantial attention in recent years compared to WGS, owing to its lower cost, shorter turnaround time, and relatively straightforward data interpretation. Crucially, tNGS exhibits superior sensitivity for detecting minor variants relative to WGS (de Diego Fuertes et al., 2026). This heightened performance is primarily attributable to the fact that tNGS leverages multiplex PCR or probe-based enrichment techniques to selectively amplify or capture specific fragments and resistance-associated loci within multiple DR-Mtb genes, which are subsequently subjected to high-throughput sequencing and bioinformatic analysis. This targeted enrichment strategy can amplify targeted microbial nucleic acids by ten-fold to several thousand-fold, thereby empowering tNGS to directly detect trace microbial targets within clinical specimens (Gou et al., 2025). A retrospective study demonstrated that tNGS achieved complete concordance with Sanger sequencing in detecting drug-resistance mutations, including HR specimens and samples wherein resistance mutations were resolved at low sequencing depths. This was particularly evident in paucibacillary specimens, validating that tNGS maintains high accuracy alongside its unique capability to detect heterogeneous mutations (Jin et al., 2025). Furthermore, alternative research indicated that regarding the “false-positive resistance” reported by Xpert Ultra, tNGS could accurately resolve these corresponding disputed mutations within the *rpoB* gene (e.g., H445N, S450W). This corroborates the capacity of tNGS to settle controversial mutations and clarify borderline resistance, thereby enabling the precise identification of HR. These robust capabilities strongly support the guidelines issued by the WHO advocating for the incorporation of tNGS into diagnostic workflows within high-burden or reference regions (Bhering et al., 2025).

### 3.3 Other emerging technologies

#### 3.3.1 Fluorescence melting curve analysis

Fluorescence Melting Curve Analysis (FMCA): Probe-based FMCA represents an efficient methodology for variant detection by monitoring the melting temperatures of probe-target hybrids. The technical premise relies on the fact that the melting temperatures of distinct mutant DNAs and wild-type DNAs differ by at least 1.86 °C, thereby enabling the reliable discrimination between drug-susceptible (wild-type) and drug-resistant (mutant) strains. Regarding HR identification, this approach is capable of resolving resistant mutations present at frequencies as low as 10%. Despite demonstrating distinct clinical potential, FMCA is constrained by significant limitations: it currently supports the interrogation of only a selective subset of resistance genes, making the expansion of its diagnostic repertoire a paramount objective for future research. Furthermore, as a targeted diagnostic modality, FMCA is inherently restricted to identifying pre-defined mutations (Chang et al., 2025).

#### 3.3.2 RNA biomarkers

Given that alterations in the transcriptome constitute one of the earliest cellular responses to drug exposure, investigators have explored the transcriptomic responses of mycobacteria under antibiotic pressure or environmental stress. Accumulating evidence reveals that analogous stressors or drugs sharing similar mechanisms of action trigger convergent transcriptomic profiles, suggesting the feasibility of leveraging RNA biomarkers for drug resistance detection (Miotto et al., 2022). Building upon this premise, Amandine et al. performed a multiplexed quantitative analysis of the transcriptional stress responses in Mtb strains following 6 hours of drug exposure. They successfully identified a suite of drug-specific mRNA biomarkers and validated their efficacy in predicting tuberculosis drug resistance, demonstrating sensitivity and specificity profiles that surpassed those of conventional pDST. However, the performance of RNA biomarker assays in detecting HR remains suboptimal; this is because the biomarker expression signatures induced by drug stress within the susceptible population only become discernible when the resistant subpopulation reaches a substantial proportion (exceeding 70%). Consequently, this high threshold renders RNA-based diagnostic platforms relatively insensitive for early HR interception (Sury et al., 2025).

#### 3.3.3 MALDI-TOF MS

Nucleic acid-based matrix-assisted laser desorption/ionization time-of-flight mass spectrometry (MALDI-TOF MS) has emerged as an efficacious diagnostic tool for the rapid and precise identification of mycobacterial species and Mtb drug-resistance profiles. This platform enables the detection of HR based on the concurrent visualization of wild-type and mutant mass spectra peaks within the generated profiles (Zhang et al., 2025). Some studies have shown that when the mutation proportion reaches 5%~25%, the nucleic acid MALDI-TOF MS can identify HR infection. Its advantages of high flux, accuracy and low cost have good application prospects in the diagnosis of clinical drug-resistant tuberculosis, especially in tuberculosis hospitals (Yang et al., 2023a). Nevertheless, current studies remain sparse and are confined to a limited selection of anti-tuberculosis agents. Larger-scale investigations across more diverse patient cohorts are warranted to comprehensively evaluate its scalability and applicability for capturing Mtb heteroresistance.

In summary, the currently available detection platforms for Mtb heteroresistance each possess distinct advantages and limitations. To assist clinicians, microbiologists, and laboratory specialists in making optimal technological selections tailored to their specific resource settings, the critical characteristics of each diagnostic platform have been systematically summarized in **Table 1** (Rigouts et al., 2019; Merker et al., 2020; Zheng et al., 2022; Danchuk et al., 2024; Lavrenchuk et al., 2025; Du et al., 2026; Park et al., 2026).

**Table 1:** Comprehensive comparative synthesis of diagnostic platforms for Mtb heteroresistance.

Diagnostic platform	LoD	Target range	Primary advantages	Major limitations	Turn around time	Clinical feasibility
Pdst	1%	N/A	Provides functional, viable evidence of resistance.	Extremely slow; easily biased by critical concentrations.	4–8 weeks	High (routinely carried out in clinical practice)
Xpert MTB/RIF Ultra/XDR	5%-60%	(primarily *rpoB* region)	Rapid; fully automated; point-of-care friendly.	Poor sensitivity for minor heteroresistant sub-clones.	1–2 hours	Extremely High (used widely around the world)
LPAs	5%	Selected core 1st & 2nd line loci	Simultaneously detects multiple major mutations.	Prone to laboratory contamination.	4–6 hours	High (Used by most countries)
ddPCR	0.1%-1%	Highly restricted to pre-set mutations	Absolute quantification of ultra-minor sub-clones.	Lacks multiplexing capacity for broad resistance profiles.	4–8 hours	Low (research-focused)
WGS	0.01%-10%	Genome-wide (comprehensive)	Provides complete phylogenetic and resistome profiles.	High cost; relies heavily on depth and complex pipelines.	3–7 days	Moderate (centralized genomics centers)
tNGS	0.01%-10%	Extensive resistance-determining regions	Superior depth for minor clones; excellent cost-benefit compromise.	Misses novel mutations outside preset panels.	2–3 days	High (rapidly expanding in hospitals)
FMCA	10%	Moderate (major resistance loci)	Closed-tube system; rapid; cost-effective.	Cannot distinguish complex overlapping mutations.	3–5 hours	Moderate (Used by few countries)
RNA Biomarkers	70%	Transcriptional expression panels	Monitors live, dynamic responses	RNA is highly unstable	1–2 days	Low (translational stage)
MALDI-TOF MS	5%-25%	Specific biomarker protein spectra	Ultra-low per-sample reagent cost; rapid processing.	Requires mass spectral libraries; low resolution for minor variants.	1–2 hours	Moderate (needs Mtb libraries)

## 4 Clinical significance of heteroresistance

### 4.1 Impact on treatment outcomes

The expansion and breakthrough growth of HR subgroups during clinical treatment are the main reasons for their evolution into complete drug resistance, leading to treatment failure. This phenomenon is more significant in other high-risk populations such as immunocompromised patients, bloodstream infection patients, and transplant recipients (Brukner and Oughton, 2025). Studies have revealed that the incidence of HR stands at 25% among patients with successful TB treatment outcomes, compared to a striking 75% in those experiencing treatment failure, strongly implying a significant correlation between HR and adverse clinical outcomes (Du et al., 2026). Concurrently, the decelerated decline in bacterial load mediated by HR prolongs the time to sputum culture conversion, thereby compromising the overall treatment success rate of tuberculosis (Martinecz et al., 2023). Furthermore, HR can persist in the majority of TB-infected patients and even continue to evolve during the course of therapy, escalating into a broader drug resistance profile—most notably with rifampicin and isoniazid HR—which concomitantly elevates the risk of disease relapse (Engelthaler et al., 2019; Chen et al., 2021a).

Conversely, conflicting evidence suggests that low-frequency HR may not substantially jeopardize therapeutic outcomes. A prior study indicated that an HR frequency of <5% in newly diagnosed TB patients exerted negligible impact on final treatment success (Shin et al., 2018). Similarly, a cohort study enrolling hundreds of drug-resistant TB patients demonstrated that individuals harboring an HR frequency between 1% and 5% experienced clinical outcomes that did not differ significantly from those without HR (Crowder et al., 2024). Mirroring these findings, identical observations have been documented for ultra-low-frequency HR (<1%) (Chen et al., 2021b).

These divergent findings inevitably provoke a critical question: Can low-frequency HR be safely cross-examined or discounted in routine clinical practice? Or, more fundamentally, what constitutes the minimum threshold of HR frequency that carries definitive clinical significance? At present, a definitive consensus remains elusive. To address this conundrum, further prospective, in-depth investigations are urgently warranted to establish a validated, explicit diagnostic threshold of HR for clinical management. Nevertheless, it is undeniable that high-frequency HR (>15%–20%) carries far greater clinical significance and deterministic risk than its low-frequency counterparts (particularly <5%).

### 4.2 Evolutionary trajectory of drug resistance

HR serves as an evolutionary bridge driving the progression of drug resistance from low to high levels, and from monoresistance to multidrug resistance. Intra-host dynamics studies indicate that during the early phase following the initiation of therapy or major modifications to treatment regimens, intra-host genetic diversity undergoes a transient increase. Under the selective pressure of drugs, the HR resistant subpopulations are progressively enriched and selected (Nimmo et al., 2020). A longitudinal tracking study spanning up to 9 years in a single patient vividly demonstrated this stepwise evolutionary process: the patient’s founding Mtb strain initially exhibited HR to isoniazid and ethambutol, accompanied by multiple concomitant *rpoB* mutations. Over time, under intense and fluctuating drug selective pressure, the ongoing selection for mutants with low fitness costs and high resistance levels triggered clonal variations and selective sweeps. This eventually led to the fixation of the most fit resistant variants, culminating in the emergence of XDR-TB (Hjort et al., 2022). Similarly, an investigation analyzing the intra-host microevolution of Mtb across a complex clinical course in a German patient documented 13 clinical isolates harvested over a 27-year treatment history. Notably, dynamic evolution accompanied by co-existing heterogeneous subpopulations was observed in 6 of these isolates. The emergence and extinction of distinct subpopulations were directly correlated with sequential modifications of the therapeutic regimens. These insights underscore that the evolution of drug resistance is fundamentally shaped by protracted, subtherapeutic, or ineffective treatment regimens, demonstrating that the fixation of resistance mutations within a bacterial population is a highly dynamic process (Sonnenkalb et al., 2021).

### 4.3 Clinical management recommendations

When HR is suspected or confirmed in clinical settings, clinicians face intricate therapeutic adjustment dilemmas. Crucially, heteroresistance-specific management pathways and frequency cut-offs have not yet been standardized within global tuberculosis guidelines, such as those issued by the WHO. Consequently, the various frequency-based thresholds proposed in current literature should not be interpreted as definitive clinical algorithms, but rather as evolving conceptual frameworks reflecting potential clinical implications based on preliminary cohort evidence. For instance, regarding rifampicin HR, available evidence highlights divergent clinical candidate strategies. On one hand, several studies suggest that the detection of rifampicin HR strongly portends an elevated risk of progression to MDR-TB, necessitating a corresponding adjustment of the therapeutic regimen to incorporate optimized second-line agents under specialist guidance (World Health Organization, 2022). On the other hand, accumulating evidence indicates that when low-frequency mutant sub-populations (e.g., a 1%–10% variant allele frequency of the S531L mutation) are present in the absence of companion first-line resistance, escalating the drug dosage (e.g., high-dose rifampicin at 30–35 mg/kg) might assist in achieving phenotypic clearance; however, this approach demands rigorous evaluation under close therapeutic drug monitoring; for fluoroquinolones, preventing the selection or enrichment of HR mediated by *gyrA* mutations often requires drug exposures equivalent to 4-to-8 times the standard dose, which mandates that clinicians stringently weigh the benefit against potential cumulative toxicity risks (Martinecz et al., 2023; Maitre et al., 2024). Similarly, regarding pivotal core and novel agents like bedaquiline and linezolid, the identification of potential HR variants should not prompt an empirical discontinuation. Instead, their clinical utilization must remain heavily contingent upon the broader individualized resistome profile, companion drug efficacy, patient tolerability, and expert multi-disciplinary consultation. Driven by the profound impact of HR on therapeutic prognoses, Previous studies have proposed the following summary of evidence table for anti-tuberculosis drug HR (**Table 2**) (Du et al., 2026):

**Table 2:** Summary of evidence for Mtb heteroresistance.

Anti-TB drug	HR frequency	Therapeutic principle
Rifampicin	Any detection (≥1%)	May portend clinical failure; consider managing as MDR-TB or utilizing companion second-line drugs under specialist consultation.
	1%-10%, with no concomitant first-line resistance	High-dose rifampicin therapy (30-35mg/kg) might suppress minor sub-clones (e.g., S531L), requires close TDM.
Isoniazid	> 10%	Suggests high risk of standard regimen failure; potential implication for dropping from standard regimens under expert guidance.
	5%-10%	High-dose isoniazid (10-15mg/kg) or enhanced adherence support may be considered to ensure adequate drug exposure.
	<5%	Standard dose treatment might still work.
Fluoroquinolones	> 5%	May compromise standard efficacy; consider substitution or tailored dose adjustments (e.g., levofloxacin 1000-1250mg).
	1%-5%	Consider initiating therapeutic drug monitoring.
	<1%	Standard dose treatment might still work.
Bedaquiline/Linezolid	Any detection	Given the limited availability of alternative regimens, a multidisciplinary consultation and a rigorous risk-benefit analysis are highly recommended before considering the continuation of its usage.

## 5 Discussion

HR represents a critical challenge in tuberculosis research, highlighting the limitations of the traditional binary (susceptible vs. resistant) diagnostic paradigm. Rather than a transient state, HR is a dynamic, multi-dimensional population phenomenon driven by both genetic variations and phenotypic adaptations, such as metabolic remodeling. This phenomenon partly explains treatment failure and relapse in patients receiving standardized chemotherapy. Translating HR research into clinical precision medicine is currently hindered by two practical challenges.

First, the clinical significance threshold remains poorly defined. While evidence indicates that high-frequency HR (>15%–20%) correlates with treatment failure and resistance escalation, studies on low-frequency HR (1%–5% or <1%) suggest it may not substantially jeopardize therapeutic outcomes. This discrepancy indicates that the intra-host trajectory of minor resistant sub-clones is determined by a balance between bacterial fitness costs and host immune clearance. Prospective, multi-center cohort studies are required to establish standardized clinical intervention thresholds and prevent suboptimal or excessive treatment.

Second, although high-sensitivity diagnostic technologies are promising, they are not universally feasible at present. Droplet digital PCR remains primarily in the research stage, while whole-genome sequencing, despite providing comprehensive resistome profiles, requires extensive bioinformatic infrastructure that constrains its deployment in decentralized or low-resource laboratories. Although targeted next-generation sequencing emerges as a practical candidate for frontline workflows due to its high enrichment capacity, accurate resolution of disputed variants (e.g., *rpoB* H445N), and superior sensitivity in paucibacillary specimens, mitigating operational costs and establishing standardized diagnostic protocols remain essential for its widespread implementation.

Looking forward, integrating high-sensitivity molecular diagnostics such as tNGS into frontline clinical algorithms represents an important pathway toward optimizing tuberculosis control. This approach facilitates earlier clinical interventions before minor resistant sub-populations transition into fixed, high-level resistance. Future research should prioritize the development of cost-effective, high-throughput multiplexed molecular detection kits and further characterize the microevolutionary dynamics of resistant sub-populations within host landscapes. Transitioning from empirical chemotherapy to quantified, heterogeneity-aware precision medicine is indispensable for improving patient outcomes and supporting global strategies for tuberculosis elimination.
